# Phenotyping Chronic Obstructive Pulmonary Disease Through Principal Component Analysis: Identification of Clinical Clusters

**DOI:** 10.7759/cureus.82811

**Published:** 2025-04-22

**Authors:** Evgeni V Mekov, Nikolay A Yanev, Nedelina Kurtelova, Teodora Mihalova, Adelina Tsakova, Yordanka Yamakova, Rosen E Petkov

**Affiliations:** 1 Department of Pulmonary Diseases, Medical University-Sofia, Sofia, BGR; 2 Central Clinical Laboratory, Medical University-Sofia, Alexandrovska University Hospital, Sofia, BGR; 3 Department of Anesthesiology and Intensive Care, Medical University-Sofia, Sofia, BGR

**Keywords:** aco, clusters, copd, personalized medicine, phenotypes, principal component analysis

## Abstract

Introduction

Chronic obstructive pulmonary disease (COPD) is a heterogeneous condition with varied clinical presentations and prognoses. Identifying patient phenotypes is essential for developing personalized treatment strategies. Principal component analysis (PCA) is a statistical method that can be employed to uncover clinical clusters and gain insight into the relationships among different disease characteristics. This study aims to analyze COPD patient phenotypes using PCA and to identify the key clinical features influencing their distribution.

Materials and methods

This was a prospective, observational outpatient study involving 96 patients diagnosed with COPD. Data collected included demographic, clinical, spirometric, echocardiographic, laboratory, and functional parameters. PCA was applied to reduce data dimensionality and to identify the principal components underlying phenotype structure.

Results

The first two principal components accounted for 62% of the total variance, underscoring the clinical heterogeneity of COPD. Visualization of the PCA revealed four distinct clusters that align with recognized COPD phenotypes: chronic bronchitis, emphysema, COPD with asthmatic features (previously referred to as asthma-COPD overlap), and the non-exacerbator type. Each cluster was associated with specific clinical characteristics.

Conclusions

PCA enabled the identification of four distinct clinical clusters among COPD patients: bronchitis, emphysema, COPD with asthmatic features, and non-exacerbator. This approach helps clarify the relationship between clinical characteristics and supports a more personalized approach to treatment.

## Introduction

Principal component analysis (PCA) is a widely used dimensionality reduction method and a fundamental tool in unsupervised machine learning and data analysis [1]. It transforms high-dimensional data into a lower-dimensional format while preserving as much of the original information as possible. This is done by identifying directions, referred to as principal components, along which the data shows the greatest variability.

Chronic obstructive pulmonary disease (COPD) is one of the leading causes of morbidity, mortality, and healthcare burden globally [2]. Between 29% and 47% of individuals with COPD experience at least one exacerbation annually [3]. The disease is characterized by significant heterogeneity in clinical features and systemic involvement, contributing to variable risks of exacerbation, hospitalization, and mortality. Phenotyping COPD patients can provide a more accurate assessment of these risks. These phenotypes vary depending on patient-specific factors such as smoking history, respiratory symptoms, comorbidities, spirometric findings, physical activity levels, and inflammatory biomarkers [4-6].

Over the years, several updates have been made to classification algorithms for COPD. Notably, the Global Initiative for Obstructive Lung Disease (GOLD) introduced the A-B-C-D classification in 2011. In 2023, GOLD revised this scheme, merging groups C and D into a single group - labeled “E” - to underscore the clinical significance of exacerbations [7].

This study aims to apply PCA in a cohort of COPD patients to identify potential clinical clusters that may reflect different disease trajectories.

## Materials and methods

Study design

This prospective observational outpatient study was conducted to investigate factors associated with COPD severity and its clinical course. All consecutive patients who met the predefined eligibility criteria and provided signed informed consent were included.

Study population

Eligible participants were adults over 40 years of age, current or former smokers with a history of more than 10 pack-years, and diagnosed with COPD based on a post-bronchodilator forced expiratory volume in one second (FEV₁)/forced vital capacity (FVC) ratio of less than 0.7. Only patients in a stable condition, without exacerbations or changes in treatment for at least one month prior to enrollment, were included.

Study measures

Demographic data such as age, sex, and smoking status were recorded. Symptom-based variables included the presence of cough, wheezing, sputum production, and shortness of breath, all reported as binary (yes/no) variables. Comorbidities were systematically documented and included a history of bronchial asthma, asthma-COPD overlap (ACO), emphysema, bronchiectasis, chronic bronchitis, obstructive sleep apnea, arterial hypertension, osteoporosis, dyslipidemia, hyperuricemia, and depression. The Charlson Comorbidity Index was also calculated.

Disease-specific assessments included post-bronchodilator spirometry measurements: FVC, FEV₁, FEV₁/FVC ratio, and peak expiratory flow. The number of moderate and severe exacerbations in the previous year was documented, with severe exacerbations defined as those requiring hospitalization or emergency department visits due to worsening respiratory symptoms.

Hematological parameters were assessed via blood samples, including CBC, CRP, fibrinogen, and α1-antitrypsin levels. Cardiovascular status was evaluated through echocardiography, which measured ejection fraction, shortening fraction, left ventricular end-diastolic and end-systolic dimensions, interventricular septal thickness, posterior wall thickness of the left ventricle, left atrial diameter and surface area, E and A wave velocities, acceleration time, ejection time, deceleration time, left atrial pressure, right ventricular free wall thickness, mean pulmonary artery pressure at rest, and maximal and minimal inferior vena cava diameters. An ECG was also performed to assess resting heart rate, the presence of atrial fibrillation, and the presence of peaked P waves (>2.5 mm) in lead II (p-pulmonale).

Diaphragmatic function was measured using thoracic ultrasound, evaluating both left and right diaphragmatic excursion at rest and following exertion. All patients performed a six-minute walking test (6MWT) to assess functional capacity, recording total distance walked and post-test peripheral oxygen saturation (SpO₂). An echocardiographic follow-up exam was conducted after the 6MWT. Dyspnea severity was assessed with the modified Medical Research Council (mMRC) dyspnea scale, and quality of life was evaluated using the COPD Assessment Test (CAT).

Statistical analysis

All collected variables were included as input for PCA. Continuous variables (e.g., FEV₁ and eosinophil count) were used in their original form, while binary variables (e.g., presence of cough and asthma history) were coded as 0 or 1. PCA was performed using the corrr package in RStudio. Prior to analysis, all variables were standardized to have a mean of 0 and an SD of 1.

A biplot was generated to visualize the relationships between input variables and their contributions to the first two principal components (Dim1 and Dim2). In this plot, each arrow represents a clinical variable; the arrow’s direction and length indicate its influence on the components. Variables pointing in the same direction are positively correlated, while those in opposite directions are negatively correlated. This visualization aids in identifying clusters of variables and their role in distinguishing different COPD phenotypes.

Ethics statement

The study was approved by the local ethics committee. All participants signed informed consent forms and received standard treatment as determined by their attending physician.

## Results

A total of 96 patients with COPD were included in the study. The mean age was 65.1 ± 8.1 years, and the mean FEV₁ was 55.8 ± 18.3%. Table 1 presents the clinical and functional characteristics of the study population.

**Table 1:** Patient and disease characteristics 6MWT, six-minute walking test; CAT, COPD Assessment Test; FEV₁, forced expiratory volume in one second; FVC, forced vital capacity; GOLD, Global Initiative for Obstructive Lung Disease; mMRC, modified Medical Research Council; PAPm, mean pulmonary arterial pressure

Characteristic (± SD)	All patients (n = 96)
Age, years	65.1 ± 8.1
Male, n (%)	60 (62.5%)
Active smokers, n (%)	33 (34.4%)
Smoking pack-years	28.5 ± 14.9
FEV₁, % predicted	55.8 ± 18.3
FVC, % predicted	77.9 ± 22.5
Distance at 6MWT, m	372 ± 110
CAT score	15.6 ± 9.2
mMRC score, n (%)	
0	11 (11.5%)
1	15 (15.6%)
2	28 (29.2%)
3	41 (42.7%)
4	1 (1%)
Moderate exacerbations (past year)	
Mean ± SD	0.73 ± 0.84
Median (IQR)	1 (0-1)
PAPm at rest, mmHg	22.4 ± 7.1
PAPm after 6MWT, mmHg	26.2 ± 8.6
Severe exacerbations (past year)	
Mean ± SD	0.97 ± 0.87
Median (IQR)	1 (0-1)
Frequent exacerbators (GOLD E), %	73 (76%)

**Figure 1:**
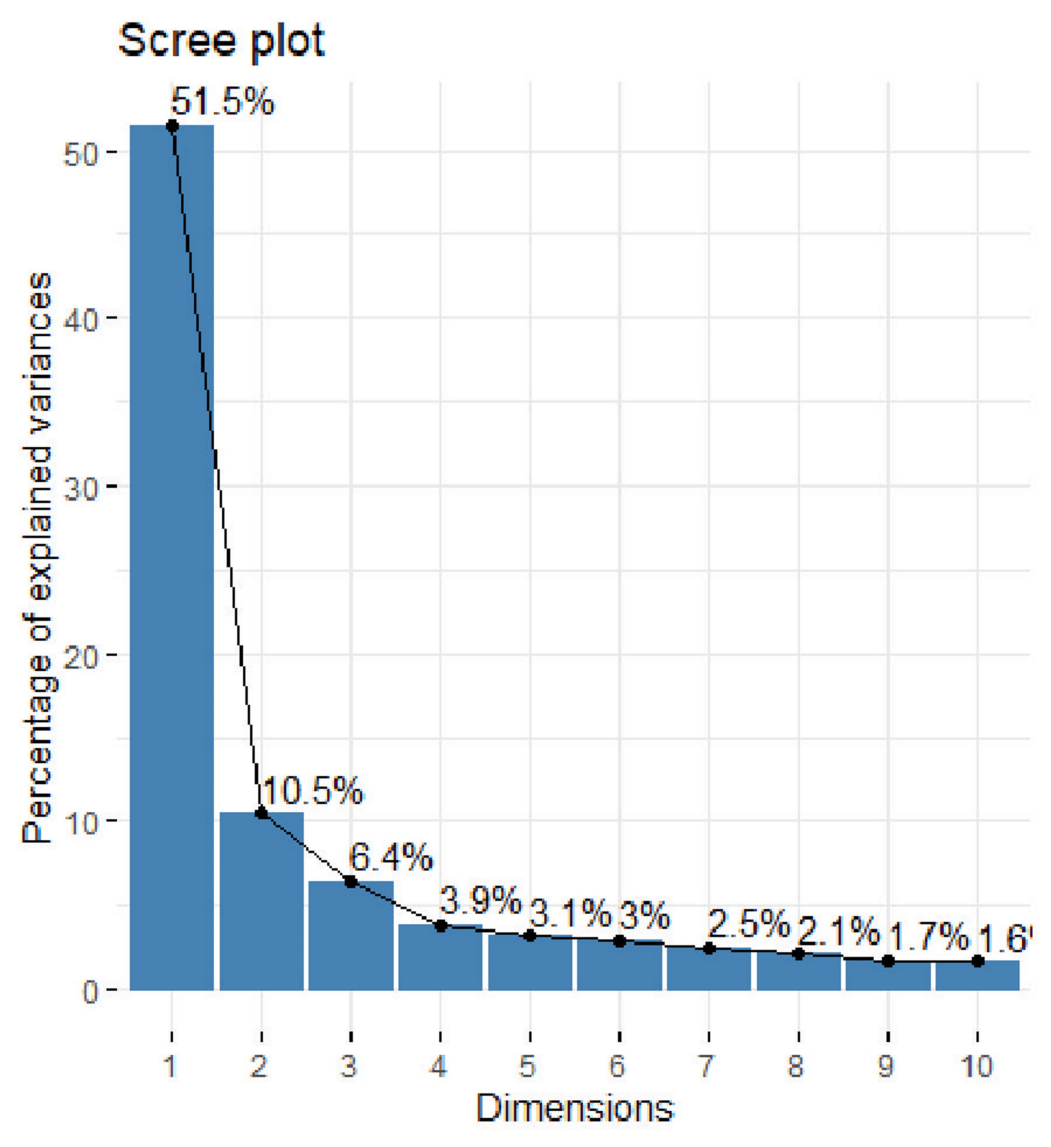
Scale of components

The results of the PCA are presented in Table 2. The “Cumulative proportion” row shows that the first principal component accounts for 51.5% of the total variance, indicating that nearly half of the dataset’s variability can be captured by this single component. The second component explains an additional 10.5% of the variance. As illustrated in Figure 1, the proportion of total variance explained by each principal component gradually decreases. Altogether, the first three components account for approximately two-thirds of the total variance in the dataset.

**Table 2:** Importance of the first five components of PCA PCA, principal component analysis

Component	1	2	3	4	5
SD	1.50	0.68	0.53	0.41	0.37
Explained variation	0.52	0.11	0.06	0.04	0.03
Cumulative proportion	0.52	0.62	0.68	0.72	0.75

Table 3 highlights the variables with the greatest contribution to the first three principal components. The first component is most strongly influenced by the BODE index, mean pulmonary arterial pressure (PAPm) at rest and after exertion, and quality of life measures. The second component is primarily driven by echocardiographic parameters, while both echocardiographic and laboratory variables contribute significantly to the third component.

**Table 3:** Matrix of the first three main components 6MWT, six-minute walking test; CAT, COPD Assessment Test; mMRC, modified Medical Research Council; PAPm, mean pulmonary arterial pressure

Element	Component 1	Component 2	Component 3
Element 1 (value)	BODE index (0.22)	Left ventricular end-systolic dimension (echocardiography) (0.27)	Ejection time at rest (echocardiography) (-0.24)
Element 2 (value)	PAPm at rest (echocardiography) (0.21)	Fractional shortening (echocardiography) (-0.27)	Ejection time after 6MWT (echocardiography) (-0.24)
Element 3 (value)	PAPm after 6MWT (echocardiography) (0.21)	Ejection fraction (echocardiography) (-0.27)	Neutrophils (%) (0.23)
Element 4 (value)	mMRC scale score (0.20)	Left atrial dimension (echocardiography) (0.25)	E wave velocity (echocardiography) (-0.20)
Element 5 (value)	CAT score (0.20)	Left atrial surface area (echocardiography) (0.21)	Eosinophils (%) (-0.20)

**Figure 2:**
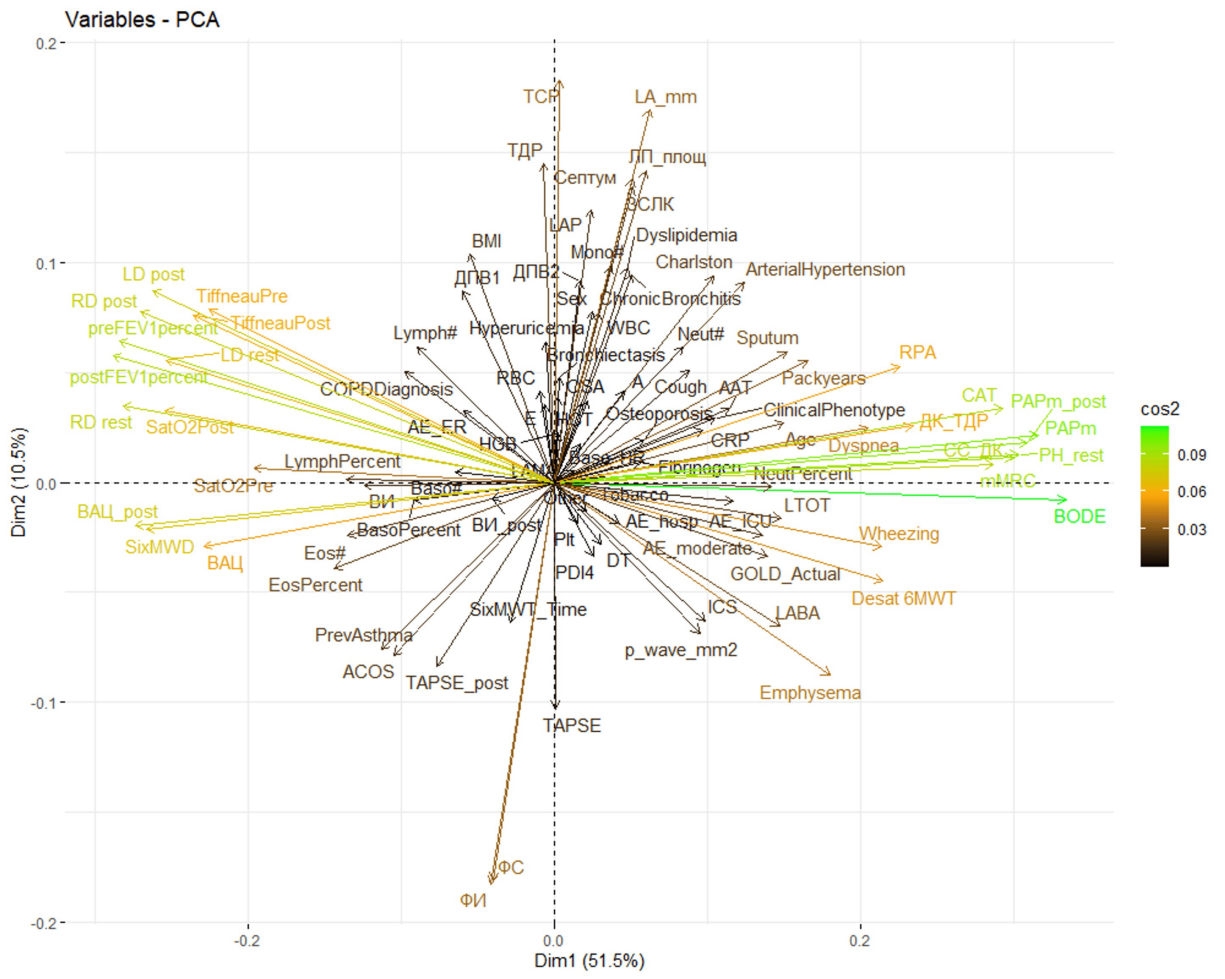
Graph of variables according to principal components Axis: The two axes, labeled “Dim1” and “Dim2,” represent the first and second principal components, respectively. The percentages (e.g., 51.5% for Dim1) indicate the proportion of total variance in the dataset explained by each component. Vectors (arrows): Each arrow represents an original variable from the dataset. The direction and length of the arrow indicate the variable’s contribution to the two principal components. Variables that are located close to each other and point in the same direction share similar profiles across the observations. Color gradient: The color gradient on the right shows the contribution or weight of each variable. Designations: Each label corresponds to a variable in the original dataset. AAT, alpha1 antitrypsin; AE_hosp, severe exacerbations in the previous year; AE_moderate, moderate exacerbations in the previous year; BODE, BODE index; Charlson, Charlson comorbidity index; COPDDiagnosis, duration (year) of COPD diagnosis; Cough, cough (symptom); Desat 6MWT, desaturation index after 6MWT; EosPercent, eosinophils (%); FИ, ejection fraction (echocardiography); ФС, shortening fraction (echocardiography); HBG, hemoglobin; HCT, hematocrit; LD post, left diaphragm mobility after 6MWT; LD rest, left diaphragm mobility at rest; LymphPercent, lymphocytes (%); Mono#, monocytes (absolute number); Neut#, neutrophils (absolute number); NeutPercent, neutrophils (%); Osteoporosis, presence of osteoporosis; PAPm, mean pressure in a. pulmonalis at rest (echocardiography); Packyears, pack-years of smoking; Plt, platelets; postFEV1percent, FEV1 (%) after bronchodilator; PrevAsthma, previous asthma diagnosis; RD post, right diaphragm mobility after 6MWT; RD rest, right diaphragm mobility at rest; SatO2Post, oxygen saturation after 6MWT; SixMWD, distance traveled at 6MWT; Sputum, expectoration (symptom); TiffneauPost, FEV1/FVC after bronchodilator; TiffneauPre, FEV1/FVC before bronchodilator; WBC, leukocytes

The influence of each characteristic on the principal components is illustrated in Figure 2. This graph (PCA biplot) displays each variable as a vector (arrow) positioned along the axes of the first two principal components (Dim1 and Dim2), which together account for the majority of the variance in the data. Higher values of the squared cosine (cos²), highlighted in green and orange, indicate the greater importance of the respective variable within the component.

Principal component 1 (Dim1), which accounted for 51.5% of the variance, is heavily influenced by the BODE index, PAPm, mMRC, and CAT. This indicates that Dim1 is primarily associated with clinical disease severity, quality of life, and pulmonary hemodynamics. Principal component 2 (Dim2), which explains an additional 10.5% of the variance, is primarily driven by echocardiographic parameters and certain laboratory markers related to the inflammatory process. Notably, distinct clusters of variables emerge in different quadrants, which helped identify four clinically meaningful COPD phenotypes: bronchitis, emphysema, COPD with asthmatic features, and non-exacerbator.

## Discussion

In this study, the principal factors influencing the clinical course of COPD were identified through PCA. The analysis revealed that the first two principal components accounted for only 62% of the cumulative variance, a relatively low value, which underscores the high heterogeneity of COPD and the numerous factors that contribute to its clinical progression [1]. Despite this, the visualization of these components revealed four distinct quadrants that correspond to different COPD phenotypes. Interestingly, these quadrants align with the traditionally recognized COPD phenotypes: bronchitis, emphysema, COPD with asthmatic features (previously known as ACO), and non-exacerbator.

In the PCA visualization, the four quadrants reflect distinct COPD phenotypes, each associated with unique clinical characteristics. The bronchitis phenotype, located in the upper right quadrant, is linked to a high prevalence of comorbidities, as indicated by the Charlson comorbidity index. These patients show impaired quality of life, as evidenced by higher CAT scores and elevated inflammatory markers, such as neutrophils. This presentation is consistent with chronic bronchitis, where airway inflammation and reduced quality of life are predominant, as shown by the vector labeled “Chronic Bronchitis” in Figure 2.

The emphysema phenotype, situated in the lower right quadrant, is characterized by frequent exacerbations, desaturation during physical exertion (such as the 6MWT), and signs of cor pulmonale, including a high P-wave on ECG. These findings are typical of emphysema, where alveolar destruction and impaired pulmonary diffusion lead to chronic hypoxemia. The “Emphysema” vector direction in Figure 2 further supports this classification.

The COPD with asthmatic features phenotype, found in the lower left quadrant, is distinguished by eosinophilia and a prior asthma diagnosis. These features suggest that individuals in this group may respond well to inhaled corticosteroids.

The non-exacerbator phenotype, located in the upper left quadrant, is characterized by fewer symptoms, better spirometric results, and a lower risk of exacerbations. This phenotype, represented by the opposite direction of the “AE_hosp” and “AE_moderate” vectors in Figure 2, corresponds to a more stable form of COPD with a more favorable long-term prognosis.

It is important to note that the opposite phenotype of the bronchitis phenotype (upper right quadrant) is not emphysema, but rather the overlap phenotype (lower left quadrant). Similarly, the opposite of the emphysema phenotype (lower right quadrant) is not bronchitis, but the non-exacerbator phenotype (upper left quadrant).

In another study, 322 COPD patients underwent PCA and cluster analysis to identify distinct clinical phenotypes [8]. Three independent components accounted for 61% of the variance, leading to the classification of subjects into four clinical phenotypes not identified by the traditional GOLD classification. Notably, patients with similar airflow limitation (FEV1) were classified into different phenotypes, demonstrating significant variations in age, symptoms, comorbidities, and predicted mortality.

The identification of four distinct COPD phenotypes, alongside the associations between clinical, functional, and echocardiographic parameters, suggests that PCA could serve as a valuable tool for phenotyping COPD patients. The separation of groups corresponds not only to well-established phenotypes but also enables an objective and statistically valid classification.

Limitations

This study has several limitations. First, the sample size was relatively small, which may limit the generalizability of the findings. Second, the study population consisted of outpatients from a single center, meaning the results may vary in different settings or in larger, multicenter studies. Third, while PCA is an effective tool for identifying patterns within data, the scarcity of similar studies makes it challenging to directly compare our results, highlighting the need for further research.

## Conclusions

PCA of COPD patients enables the identification of four distinct clusters that correspond to well-established disease phenotypes: bronchitis, emphysema, COPD with asthmatic features, and non-exacerbator. By using statistical methods to distinguish these phenotypes, it becomes possible to identify the characteristics that are most strongly related to each other. This approach facilitates a more personalized treatment strategy tailored to the specific phenotype of each patient.

## References

[1] Jolliffe IT, Cadima J (2016). Principal component analysis: a review and recent developments. Philos Trans A Math Phys Eng Sci.

[2] Chen S, Kuhn M, Prettner K (2023). The global economic burden of chronic obstructive pulmonary disease for 204 countries and territories in 2020-50: a health-augmented macroeconomic modelling study. Lancet Glob Health.

[3] Vestbo J, Anderson W, Coxson HO (2008). Evaluation of COPD Longitudinally to Identify Predictive Surrogate End-points (ECLIPSE). Eur Respir J.

[4] Burgel PR, Paillasseur JL, Roche N (2014). Identification of clinical phenotypes using cluster analyses in COPD patients with multiple comorbidities. Biomed Res Int.

[5] Rennard SI, Locantore N, Delafont B (2015). Identification of five chronic obstructive pulmonary disease subgroups with different prognoses in the ECLIPSE cohort using cluster analysis. Ann Am Thorac Soc.

[6] Kim S, Lim MN, Hong Y, Han SS, Lee SJ, Kim WJ (2017). A cluster analysis of chronic obstructive pulmonary disease in dusty areas cohort identified three subgroups. BMC Pulm Med.

[7] (2023). Global Strategy for Prevention, Diagnosis and Management of COPD: 2023 Report. COPD.

[8] Burgel PR, Paillasseur JL, Caillaud D (2010). Clinical COPD phenotypes: a novel approach using principal component and cluster analyses. Eur Respir J.

